# Evaluation of Yellow Mealworm (*Tenebrio molitor*) Larvae Meal as a Carbon Source in Biofloc System for Dwarf Crayfish (*Cambarellus montezumae*) Culture

**DOI:** 10.1155/anu/1521301

**Published:** 2025-08-06

**Authors:** Reyna Perla Paredes-Chávez, José Antonio Mata-Sotres, Rocío Parra-Laca, José Alberto Ramírez-Torrez, Daniel Becerril-Cortés, María del Carmen Monroy Dosta

**Affiliations:** ^1^Man and His Environment Department, Metropolitan Autonomous University Xochimilco Unit, Calzada del Hueso, 1100 C.P. 04960, Coyoacan, Mexico; ^2^Science and Technology Faculty, Simon Bolivar México, Av., Rio Mixcoac N° 48, Col. Insurgentes Mixcoac, Benito Juarez C.P. 03920, Mexico, Mexico

**Keywords:** biofloc, *Cambarellus montezumae*, carbon source, *Tenebrio molitor*

## Abstract

The aim of this study was to evaluate the use of *Tenebrio molitor* larvae meal as a carbon source in a biofloc system (BFT) to optimize the culture of *C. montezumae*. A trial was conducted for 24 weeks, 500 *T. molitor* larvae were reared with wheat bran as substrate, and hydration once a week; at the end, 9000 *T. molitor* larvae were obtained, which were processed to make meal. *C. montezumae* was conditioned for 2 weeks in two 100 L polyethylene tanks in clear water and fed with Tropical shrimp sticks until the beginning of the experiment. A 12-week experiment was performed with three treatments and three replicates each: control (no biofloc), biofloc with molasses (BFT-M), and biofloc with *T. molitor* meal (BFT-T) as carbon source, at a 20:1 C/N ratio, adjusted every 15 days based on the feed ratio. The culture parameters of the water during the experimental period varied, but they always remained within the range required for the species. No significant differences were observed in weight gain (WG) among the three treatments (*p*  > 0.05), but the crayfish cultured in the treatments with biofloc obtained the greatest WG and the lowest feed conversion ratio (FCR). In the biofloc, nine phyla of zooplankton were identified, of which 16 genera were present in BFT-M and 13 in BFT-T. The chitinolytic bacteria showed a greater diversity and richness in BFT-T compared to the BFT-M and control. *Pseudomonas luteola* was the dominant species, and *Rhizobium radiobacter* the second one. The nutritional composition (lipids, carbohydrates, fiber, ash, and moisture) of *T. molitor* as well as biofloc from the BFT-M and BFT-T showed significant differences (*p*  < 0.05), but not in protein levels. This study demonstrated that *T. molitor* meal is a viable carbon source in *C. montezumae* biofloc culture, improving biofloc nutritional quality through increased chitinolytic bacterial diversity.

## 1. Introduction

In recent years, the aquaculture industry has innovated with the search for sustainable production techniques such as the biofloc system (BFT) which contributes to the production of freshwater and saltwater fish (i.e., *Carassius auratus*, *O. niloticus*, *Dicentrarchus labrax*, *Scophthalmus maximus*, *Takifugu rubripes*, *Salmo salar*) and crustaceans (i.e., *Litopenaeus vannamei*, *Penaeus monodon*, *Macrobrachium rosenbergii*) with less use of water, fishmeal, energy, and without the generation of polluting effluents [1–4]. The nutrient waste generated from unconsumed food, fecal materials, and metabolic by-products excreted from the cultured organism is converted into beneficial microbial biomass that can be used again by the cultured organisms [1, 3, 5]. Where the addition of an external carbon source is a conditioning that favors the microbial community to transform waste into microbial protein, nutrients, and antimicrobial metabolites, positively impacting the productive and environmental parameters of the cultivated species [5–7].

Although in recent years BFT systems have been widely used with the addition of different carbon sources (i.e., molasses, sugar, acetate, starch, etc.), it is a reality that the aquaculture industry needs more sustainable and locally sourced alternatives [8–10].

Insect meal is currently seen as a new ingredient with great potential for aquaculture, as in many regions of the world is an inexpensive resource, easy to culture, that can be exploited [11–13].

In particular, *Tenebrio molitor* is one of the most widely used insects as it has certain advantages such as a high reproductive rate, low production cost, and ease of cultivation [12, 14–16]. Furthermore, *T. molitor* meal has been reported to improve the growth and immunity of fish and crustaceans [15]. Likewise, *T. molitor* meal has a high chitin content that can be used in the production of various crustaceans [14].

The crayfish *Cambarellus montezumae* is a species with great potential for cultivation in BFT systems since it has been proven that its growth is adequate in high densities, large amounts of sediment, and microorganisms within the medium [17–19]. Therefore, the culture of *C. montezumae* is important both for the generation of low-cost animal protein and for its conservation, due to the constant impact on their natural habitat, pollution, overfishing, and the introduction of exotic species [20]. In relation to BFT culture, insect meal, including *Tenebrio*, has only been used as a replacement for fish meal in the diets of cultured organisms but not as a carbon source in BFTs [8, 20, 21].

Although the popularity of molasses, wheat flour, and biodegradable polymers to produce aquatic organisms in Biofloc, further research is needed on the use of low-cost and accessible nonconventional alternative sources. Hence, the aim of this research was to evaluate the use of *T. molitor* larval meal as a carbon source in the BFT system to optimize the culture of *C. montezumae*.

## 2. Materials and Methods

### 2.1. *T. molitor* Meal Procedure

Five hundred *T. molitor* larvae were maintained for 24 weeks in an 18 × 31 × 44 cm container at an average temperature of 25°C, average relative humidity of 55% in a wheat bran substrate, providing carrot as a source of hydration once a week [16]. At the end of the culture, 9000 larvae of 7-week-old were obtained, which were subjected to drying, according to Quintero [22] (Thermolyne Series 9000 Oven, USA), pulverizing (T-Fal MF6021MX, France), and sifted to 0.5 mm to make meal.

### 2.2. Rearing Conditions

Two hundred *C. montezumae* were donated from a local producer in México city and transported to the Laboratory of Chemical Analysis of Live Food of the Universidad Autónoma Metropolitana-Xochimilco. Crayfish were acclimatized for 2 weeks in two 100 L polyethylene tanks in clear water and fed with commercial diets (Tropical shrimp sticks, 45% crude protein and 7% lipids) until the beginning of the experiment.

### 2.3. Culture Conditions

A 12-week trial was performed with three different treatments: control (clear water), biofloc treatment with the addition of molasses (BFT-M), and biofloc with the addition of *T. molitor* larva meal (BFT-T) as a carbon source, with a density of 80 org *m*^2–1^ [18]. In both cases, since day one until the end of the trial, carbon source was added daily in a 20:1 C/N ratio to ensure the proper formation of flocs, based on exigency calculations according to Avnimelech [23], adjusted every 15 days in function of biomass. Each treatment was performed in triplicate.

The cultured organisms were randomly distributed in 12 plastic ponds at 75% capacity (80 L), at a density of 20 crayfish per tank [18], and the crayfish were fasted 12 h before the initial measure, with an average initial weight of 0.8 ± 0.06 g and 3.1 ± 0.12 cm of length. Crayfish were fed every third day at a rate of 10% biomass with a commercial diet with a content of 45% protein, 7% lipids, 4% fiber, and 8% moisture, with a particle size of 1.5–2.0 mm (Tropical shrimp sticks, Chorzów, Poland). Silvercup feed, El Pedregal, Toluca, State of Mexico, Mexico. It was adjusted every 16 days, according to biomass.

In biofloc treatments, aeration was permanent to keep organic matter in suspension, freshwater was added regularly to compensate for the evaporation loss, and the temperature was kept at 21°C. While maintaining the quality of the control treatment water, weekly water changes of 50% were made, and the organic matter that was at the bottom of the pond was siphoned. In addition, in all tanks, 61-inch PVC pipes were placed in each tank as a form of protection and refuge for the organisms.

### 2.4. Water Quality Monitoring

The pH, temperature, and dissolved oxygen (DO) values were monitored weekly, using a Hanna model HI 9829 multiparametric meter. The total ammonia nitrogen (TAN), nitrite, and nitrate levels were measured every 10 days using a Hanna auto-analyzer Aquaculture Photometer (model HI83203) following the manufacturer's procedures. Settleable solids (SS) were measured once a week by pouring 1 L of water from the tank for 30 min into an Imhoff cone [24].

### 2.5. Sampling

At the end of the 12-week trial, crayfish were counted and weighed.

Weight gain (WG), feed conversion ratio (FCR), and survival rate (SR) were calculated as follows:

  $$\begin{array}{c} \text{WG }\left(\text{g}\right)=\text{ final weight}-\text{initial weight}, \end{array}$$

  $$\begin{array}{c} \text{FCR}=\text{ feed intake}/\text{weight gain}, \end{array}$$

  $$\begin{array}{c} \text{SR }\left(\%\right)=\text{ total number of fish harvested}/\text{total number of fish stocked}\times \;100. \end{array}$$

### 2.6. Biofloc Composition

Every 2 weeks, a sample of 100 mL was taken and allowed to settle for half an hour. Then, a 1 mL aliquot was obtained from settled solids and observed directly under the microscope at 20X and 40X (Olympus CX31, Tokyo, Japan) [25]. The taxonomic identification of the observed zooplanktonic groups was carried out according to Aladrado-Lubel [26].

### 2.7. Chitinolytic Bacteria

For the identification of chitinolytic bacteria, 100 mL of water samples were collected from all BFT-M and BFT-T tanks and diluted 1:10 in saline solution (0.9%). After this, 1 mL of each sample was inoculated in triplicate in petri dishes with bacteriological agar medium enriched with chitin and incubated at 32°C for 72 h. After the incubation period, the strains were purified by successive reseeding, and their purity and cellular morphology were subsequently verified by Gram staining.

Bacterial identification was performed using miniaturized API metabolic tests (bioMérieux, Lyon, France) and the APIWEB service.

### 2.8. Proximate Composition of Biofloc and *T. molitor* Meal

Flocs from each experimental treatment, along with mealworm meal, were analyzed in triplicate following AOAC [27]. The flocs from each BFT tank were collected using a 10 μm mesh sieve. The collected flocs were dehydrated in a steam oven (Thermolyne Series 9000 Oven, USA) at 60°C for 24 h, ground using a blender, and stored at room temperature.

Moisture and dry matter content were determined by weight difference. Ground samples were dried at 60°C for 24 h and then carbonized in a muffle furnace at 550°C for 6 h. Crude protein was determined by the micro Kjeldahl method, and nitrogen content was converted to protein using a factor of 6.25 (%N × 6.25). Total lipid was analyzed by the Soxhlet method according to AOAC [27], with petroleum ether as the extraction solvent. Carbohydrates were determined using the phenol–sulfuric acid technique. Fiber content was analyzed using acid and alkaline hydrolysis methods, and ash content was determined by calcination in a muffle furnace.

### 2.9. Statistical Analysis

The statistical assumptions of normality and homogeneity were calculated using Shapiro–Wilks and Bartlett tests, respectively [28]. A one-way ANOVA (*α* 0.05) was performed, and a Tukey (*α* 0.05) rank test was realized using the IBM SPSS Statistics 25 (IBM Corp., Armonk, NY, USA).

## 3. Results

### 3.1. Water Quality Parameters

**Figure 1:**
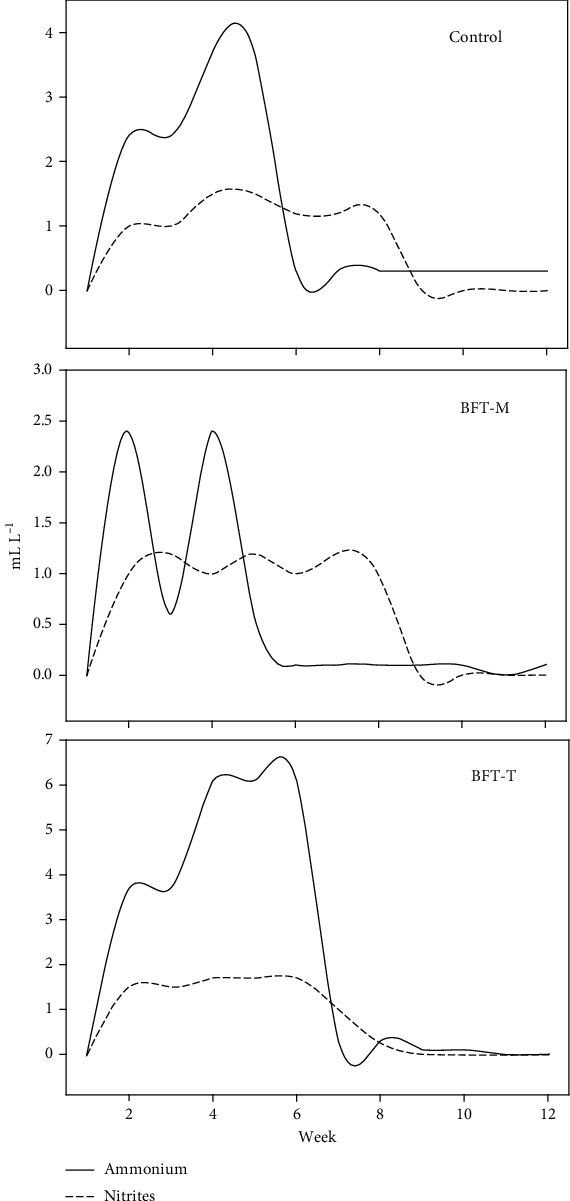
Variations in ammonium and nitrites during the experiment.

The physicochemical parameters fluctuated based on treatment conditions (Table 1). Nonetheless, they consistently adhered to the necessary range for the species, despite the absence of water exchanges in the BFT-M and BFT-T treatments. In control treatment and BFT-M, ammonium and nitrites varied initially before stabilizing, whereas in BFT-T, significant reductions occurred only by week seven (Figure 1).

### 3.2. Performance Parameters of *C. montezumae*

No significant differences (*p*  > 0.05) were detected in WG increment across the three experimental conditions, as well as in the FCR. Conversely, the SR was significantly elevated in the control group when juxtaposed with the BFT-M and BFT-T groups (Table 2).

### 3.3. Diversity of Zooplankton Associated With Flocs

**Figure 2:**
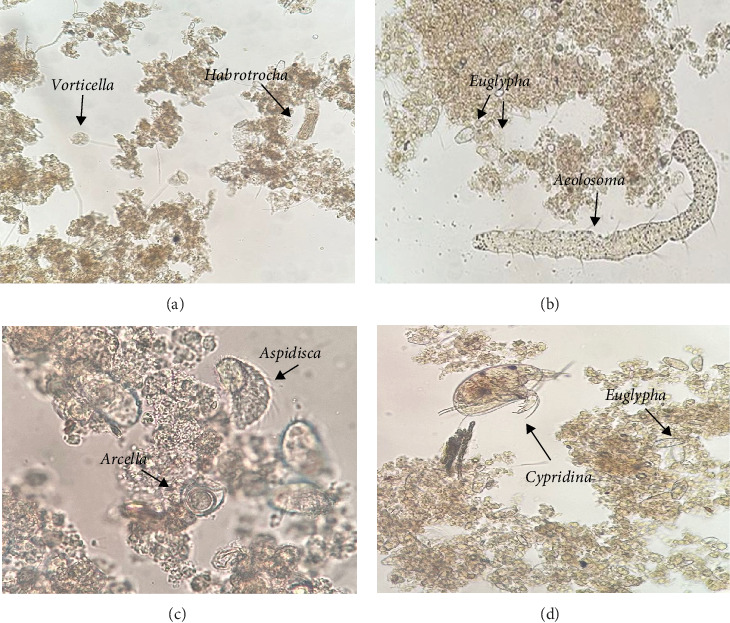
BFT-M bioflocs. (a) *Vorticella* and *Habrotrocha* (10X), (b) *Euglypha* and *Aeolosoma* (10X), (c) *Aspidisca* and *Arcella* (40X), and (d) *Cypridina* and *Euglypha* (10X).

**Figure 3:**
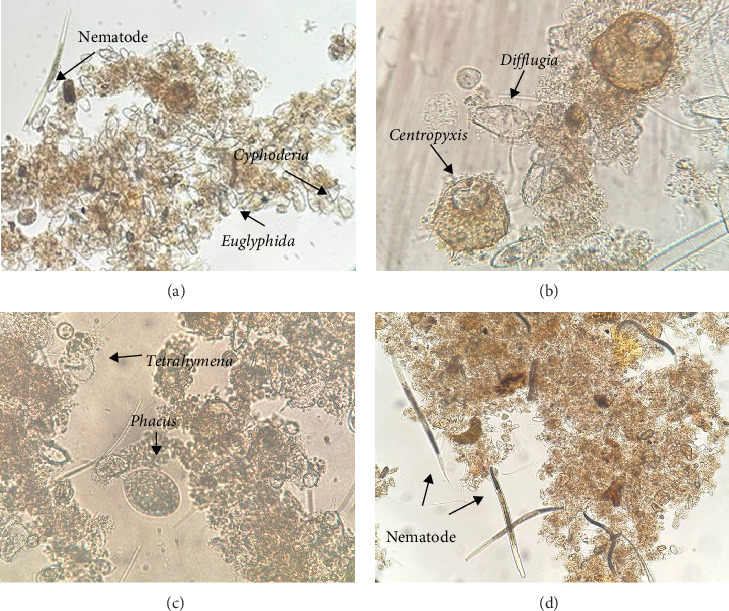
BFT-T bioflocs. (a) Nematode, *Cyphoderia* y *Euglyphida* (10X), (b) *Centropyxis* y *Difflugia* (40X), (c) *Phacus* y *Tetrahymena* (40X), and (d) nematode (40X).

In the Biofloc treatments, phyla nine were identified, within which 18 genera were identified in the BFT-M treatment and 14 genera in BFT-T (Table 3; Figures 2 and 3).

### 3.4. Chitinolytic Bacteria

**Figure 4:**
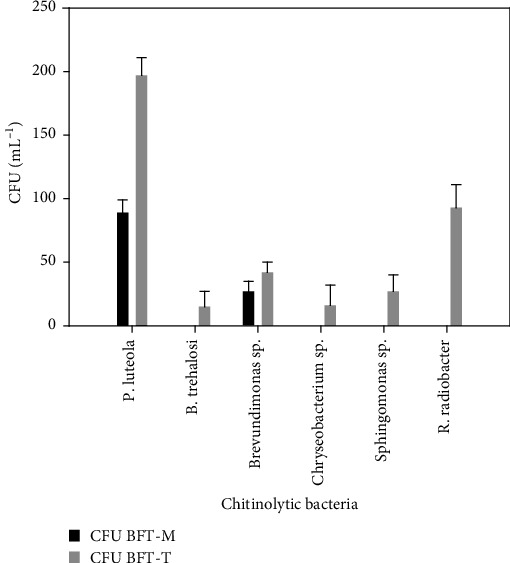
Presence and abundance (CFU) of chitinolytic bacteria isolated in BFT-M and BFT-T.

Regarding the chitinolytic bacteria identified in the biofloc treatments, there is a greater diversity and abundance of species in BFT-T compared to BFT-M and control treatments. *Pseudomonas luteola* was the species with the highest abundance, followed by *Rhizobium radiobacter* (Figure 4).

### 3.5. Proximate Composition of Biofloc and *T. molitor* Meal

In relation to the nutritional composition of *T. molitor* meal as well as flocs from the BFT-M and BFT-T treatments (Table 4). Lipid, fiber and ash contents had higher percentages in BFT-T, while carbohydrates were higher in BFT-M. In all cases, significant differences were obtained (*p*  < 0.05). However, protein is the only nutrient that did not show significant differences for any treatment (*p*  > 0.05).

## 4. Discussion

Several studies indicate biofloc technology has a significant effect on improving the water quality of fish and crustacean farming, but there are various factors that influence maintaining its good condition, such as light intensity, salinity, phytoplankton, but the most important factor is the carbon source. Easily assimilated carbohydrates enhance carbon availability for heterotrophic bacteria, facilitating improved metabolism of ammonium and nitrogen compounds. In the present study, it was observed that nitrogen compounds remained within the ranges required for crayfish cultivation [20, 21].

An increase in ammonium was noted in the BFT-T treatment during the initial 5 weeks due to the utilized carbon source. The chitin in insect meal's high molecular complexity necessitates bacterial secretion of chitinases for transformation and hydrolysis [29]. However, its development is slower within the culture compared to BFT-M, due to molasses being a simple carbohydrate easily assimilated by microorganisms in biofloc [30]. This behavior has been reported by Llario et al. [31], who noted that NH_4_^+^ concentrations peak in the initial weeks of biofloc culture during the maturation stage, wherein microorganisms, especially heterotrophic bacteria and picoplankton, proliferate and consume carbon from dissolved organic matter generated in the system [32].

Similarly, Ebeling and Timmos [33] reported that nitrifying bacteria need around 30 days to establish themselves in the culture. This agrees with what was seen in the BFT-M system, since from week four the levels of nitrites (NO_2_^−^) increased as the NH_4_^+^ decreased, and subsequently both nitrogen compounds could be maintained at low concentrations without the need for water exchange. Likewise, Monroy et al. [34] have mentioned that within the maturation stage, there is the development of chitinolytic, amylolytic, and cellulolytic bacteria that are responsible for degrading the waste of the cultivated organisms. Where, together with the presence of nematodes, rotifers, ciliates, and amebas, a control of nitrogenous waste is achieved [35]. Process that can be measured indirectly through the SS which verified that from 5 mL L^−1^ the heterotrophic communities are established [10], which agrees with what was observed at the end of the experiment, where the SS levels of both BFT exceeded 10 mL L^−1^.

In relation to the productive parameters, such as growth and FCR, no significant differences were observed between the biofloc and control treatments, where the crayfish cultured in the biofloc treatments obtained the highest WG and the lowest FCR. This could be due that the flocs were available as food 24 h a day in the system compared to the control group that only had the feed supplied as its only nutritional resource. It is important to mention that the FCR in *C. montezumae* is very variable due to its feeding habits, since it uses its pincers to hold the feed, with which it can cut the feed into pieces [36] and consequently not ingest the entire ration, leaving the rest in the tank. However, in a BFT system, this food can be incorporated back into the food web through the formation of bioflocs [37].

Conversely, the control treatment yielded superior survival outcomes compared to BFT treatments. This may stem from the maturation of the system, during which NH_4_^+^ levels escalated [20], as mortality ceased after the sixth week when nitrogen compounds were fully controlled. Nevertheless, it is crucial to acknowledge that this investigation commenced from a baseline, necessitating assessment through prior inoculation to mitigate the maturation duration and establish control over physical–chemical parameters from the beginning of the culture [6].

Regarding zooplankton diversity in BFT-M and BFT-T, in both systems, the presence of rotifers, nematodes, ciliates, and amebas predominated, being microorganisms associated with biofloc previously reported [33, 38]. Organisms that mitigate ammonia in ponds and serve as high-quality protein sources for cultured species [2].

Rotifers can consume bacteri, and the mucilage produced by their excretions helps in the formation of new flocs [13]. In addition, nematodes represent a very important food source for cultured organisms due to their high nutritional value [10]. Ciliates play an important role in the food web, since the abundance of nematodes in a BFT system is determined by the presence of ciliates, which serve as food for them, while ciliates control microalgae populations [16], maintaining the predominance of the heterotrophic community in the system. Finally, both naked and testate amebae have a high performance in the efficient removal of nitrogen present in the water [39].

The chitinolytic bacteria isolated in this work demonstrate that chitin could be effectively hydrolyzed within the BFT-T system, since all of them have been reported with chitinolytic activity for the use of this component as a source of carbon and nitrogen [29]. In addition, they have the ability to form exopolysaccharide biofilm to rapidly degrade and hydrolyze chitin by direct contact [1]. The prevalence and quantity of these bacteria were dominant in BFT-T, linked to the utilized carbon source. In contrast, BFT-M exhibited only two identified species, likely arising from the degradation of *C. montezumae* molts in the water column. In this study, *P. luteola* was identified, a bacterium that has been reported to have chitinolytic activity. In addition to being the species that was present in both BFT systems and had the highest abundance. Likewise, *Brevundimonas sp*. was present in both cultures since, in addition to its hydrolytic activity towards chitin, it also has a high denitrification activity [40]. In the case of BFT-T, the species *R. radiobacter* was most abundant after *P. luteola*, being indispensable in the system, since it has been reported to present extracellular enzymes that rapidly hydrolyze chitin [20, 41]. Like the genus *Sphingomonas sp*. and *Corynebacterium sp.*, which can degrade it into simple monomers (amino sugars) so that they can be used as a carbon source [42]. While *B. trehalosi* has been associated with respiratory diseases in ruminants, its pathogenesis mechanisms remain uncertain [42], and there is also no evidence of triggering infectious processes in *Cambarellus sp*. So, it could be intervening in some chitin degradation process or in the nitrogen cycle; however, its study needs to be expanded in order to explain its presence in the BFT-M treatment.

The results obtained from the proximate analyses of lipids and fiber in *T. molitor* meal were higher than those previously, and in contrast to the lower protein levels than what has been reported [43–45]. Which may be due to the nutritional value of the *T. molitor* larvae, which is not chemically constant since its nutritional properties depend on its feeding, culture conditions, growth phase, and the meal processing itself [46]. On the other hand, the high levels of lipids in *T. molitor* meal could be due to the meal was made with larvae that were in the last larval stage, a stage of development where maximum concentration levels for the larvae have been reported [47, 48]. This same increase for the last phase of larval development has also been reported for fiber levels [49]. Without forgetting that the fiber levels of the meal refer to the chitin content of *T. molitor*, since fiber is considered a complex carbohydrate [50]. In addition, this content may be affected by the meal production process, since the grinding of the larvae into very small particles can reduce their content, as well as high drying temperatures [51]. However, in this case, the meal was produced at low temperatures, so the values were higher than those reported.

On the other hand, the proximate composition of bioflocs is directly related to the nutritional contribution of microbial and planktonic communities, the carbon source used, and the cultured species, so their nutritional properties are usually very dynamic [10]. The protein results of BFT-M and BFT-T are considered acceptable if it is considered that most aquaculture species require a protein level between 20% and 50% in their diet [52–55]. The protein and lipid values in this research were higher than those reported by Hosain [56], Peréz-Velasco et al. [57], and Ekasari et al. [58] in the biofloc culture of the crustacean *Macrobrachium rosenbergii*. This may be related to the abundance of nematodes observed, since they have a high protein content [59] as well as the abundance of rotifers [60]. While the high lipid levels in both BFT systems could be due to the presence of ciliates and diatoms, which are a source of essential fatty acids and lipids [10], that were also observed within the experiment. It is important to mention that the high lipid content could have favored the reproduction of the organisms, because in both BFT systems, ovigerous females and hatched larvae were found, with lipids being the main source of energy used for reproduction [61]. On the other hand, fiber values were significantly higher in the BFT-T system than in BFT-M, since the carbon source has a direct effect on the fiber content of the flocs, since when cereal and vegetable flours are used, the fiber content is usually higher [62].

Regarding the ash values, they are within the range reported in crustacean biofloc [58, 60, 63], where the ash content in biofloc is related to the abundance of diatoms and microalgae since their cell walls contain silicate (an important mineral for the construction of the exoskeleton of crustaceans) [63]. While carbohydrates were found below the levels reported by Ekasari et al. [58], this could be due to the fact that they were used by heterotrophic bacteria, and although carbohydrates represent the main source of energy, if the levels of lipids and protein are optimal, the organism can grow correctly [64].

## 5. Conclusion

In conclusion, the addition of *T. molitor* meal as a carbon source in *C. montezumae* biofloc culture is a viable alternative since it produces a biofloc rich in chitinolytic bacteria that significantly improves the nutritional quality of the biofloc. Although the maturation period of the system must be considered, it is recommended to inoculate a biofloc previously developed with insect meal to reduce fluctuations in nitrogen compounds and ensure greater benefit in zootechnical parameters.

## Figures and Tables

**Table 1:** Average parameters of the different parameters throughout the experiment (mean ± SE).

Treatment	DO (mg L^−1^)	Temperature (°C)	pH	TAN (mg L^−1^)	Nitrites (mg L^−1^)	SS (mL L^−1^)
Control	4.6 ± 0.1	20 ± 1.2	7.6 ± 0.4	1.9 ± 1.4	1.2 ± 0.6	—
BFT-M	5.1 ± 0.1	20 ± 1.0	8.2 ± 0.8	1.2 ± 0.8	1.1 ± 0.5	20 mL L^−1^
BFT-T	5.0 ± 0.2	20 ± 1.2	8.1 ± 0.8	3.1 ± 2.7	0.9 ± 0.8	15 mL L^−1^
Optimal ranges⁣^*a*^	3.8 – 6.0	17 – 22	8.0 – 9.0	0.04 – 3.8	0.01 – 1.26	>5 m L^−1^ <30 mL L^−1^

Abbreviations: DO, dissolved oxygen; SS, settleable solids; TAN, total ammonia compounds.

⁣^*a*^[20, 29].

**Table 2:** WG, FCR, and survival of *C. montezumae* at the end of the experiment (mean ± SE).

Treatment	WG (g)	FCR	Survival (%)
Control	0.43 ± 0.24	1.60 ± 0.36	87
BFT-M	0.48 ± 0.06	1.43 ± 0.66	81
BFT-T	0.45 ± 0.18	1.52 ± 0.56	60

**Table 3:** Microorganisms identified in BFT-M and BFT.

Phylum	Géneros presentes en BFT-M	Géneros presentes en BFT-T
Amoebozoa	*Arcella* *Centropyxis* *Cyphoderia* *Difflugia* *Euglyphida*	*Centropyxis* *Cyphoderia* *Difflugia* *Euglyphida*

Ciliophora	*Aspidisca* *Paramecium* *Tetrahymena* *Vorticella*	*Carchesium* *Paramecium* *Tetrahymena*

Heliozoa	*Acanthocystis*	*Acanthocystis*

Rotifera	*Habrotrochapo* *Colurella* *Lecane* *Philodina*	*Cephalodella* *Colurella* *Lecane*

Nematoda	*Monhysterida*	*Monhysterida*

Euglenozoa	—	*Phacus*

Annelida	*Aeolosoma*	*Aeolosoma*

Gastrotricha	*Chaetonotus*	—

Arthropoda	*Cypridina*	—

**Table 4:** Proximate composition of biofloc and *T. molitor* meal (mean ± SE).

Proximate composition (%) on dry matter basis				
Parameters	*T. molitor* meal	BFT-M	BFT-T	*p*-Value
Protein	39.42 ± 0.51	34.30 ± 1.71^a^	33.80 ± 0.40^a^	0.65
Lipids	43.15 ± 0.24	7.92 ± 0.06^a^	10.08 ± 0.04^b^	0.001
Carbohydrates	12.52 ± 0.13	6.24 ± 0.15^a^	5.92 ± 0.04^b^	0.025
Fiber	8.41 ± 0.08	13.11 ± 0.15^a^	17.09 ± 0.35^b^	0.001
Ash	7.07 ± 0.11	19.43 ± 0.17^a^	23.11 ± 0.27^b^	0.002
Moisture	3.38 ± 0.10	7.62 ± 0.06^a^	6.25 ± 0.30^b^	0.001

*Note:* Different superscripts indicate significantly different values (*p*  < 0.05).

## Data Availability

Data sharing is not applicable to this article as no datasets were generated or analysed during the current study.
